# Supplementary material for: Humoral Immune Response to CoronaVac in Turkish Adults
Source: Vaccines (Basel). 2023 Jan 18;11(2):216. doi: 10.3390/vaccines11020216 (PMC9967599; doi:10.3390/vaccines11020216)
Supplement: Supplementary file 1 [file vaccines-11-00216-s001.zip › vaccines-2083377-supplementary/Supplementary Figure S1.pdf]

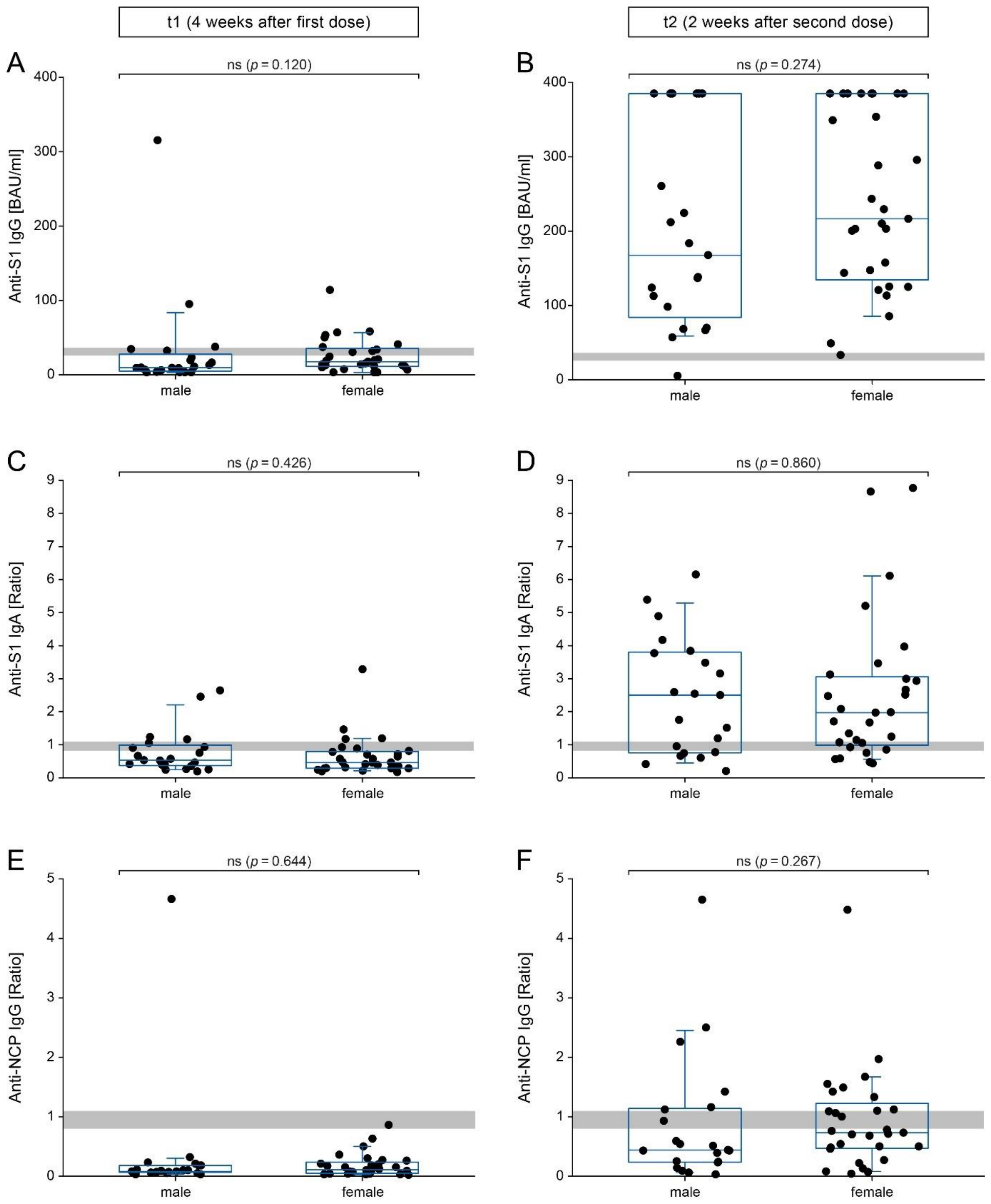

Figure S1. Antibody levels in men and women after the first and second CoronaVac dose. Anti-S1 IgG (A, B), anti-S1 IgA (C, D) and anti-NCP IgG (E, F) reactivity in 21 males and 29 females at t1 (A, C, E) and t2 (B, D, F). Data are presented as scatter plots, boxes indicate interquartile ranges (outer bounds) and medians (midlines), Whiskers present the 90th and 10th percentiles. Borderline ranges are shaded in grey. The Mann-Whitney *U* test was used to analyze differences between the sexes (ns, not statistically significant). For anti-S1 IgG, samples with results above the measurement range (>384 BAU/ml) are indicated as data points at 385 BAU/ml.
