# Supplementary material for: Humoral Immune Response to CoronaVac in Turkish Adults
Source: Vaccines (Basel). 2023 Jan 18;11(2):216. doi: 10.3390/vaccines11020216 (PMC9967599; doi:10.3390/vaccines11020216)
Supplement: Supplementary file 1 [file vaccines-11-00216-s001.zip › vaccines-2083377-supplementary/Supplementary Table S1.pdf]

Table S1. Antibody reactivity after vaccination with ChAdOx1 nCoV-19. Median titers and positivity rates of SARS-CoV-2-specific antibodies in 10 Bangladeshi adults (5 males, 5 females; age 38.4±11.7 years, range: 23-60 years, median: 37.5 years; without previous SARS-CoV-2 infection) before and after administration of two doses of ChAdOx1 nCoV-19 vaccine.

Sampling time	Anti-S1 IgG			Anti-S1 IgA		
	**Median [range]**	**Rate (n/N)[a]**	**95% CI**	**Median [range]**	**Rate (n/N)[a]**	**95% CI**
t0 (baseline pre-vaccination)	<3.2 BAU/ml [<3.2-7.3]	0% (0/10)	0-32.1%	Ratio 0.2 [0.1-0.9]	0% (0/10)	0-32.1%
t1 (19-21 days after first dose)	143.7 BAU/ml [24.0-565.6]	90.0% (9/10)	57.4-99.9%	Ratio 2.0 [0.6-9.0]	90.0% (9/10)	57.4-99.9%
t2 (19-20 days after second dose)	807.2 BAU/ml [431.0-1523.4]	100% (10/10)	67.9-100%	Ratio 1.7 [0.7-7.0]	90.0% (9/10)	57.4-99.9%

Abbreviations: BAU/ml, binding antibody units per milliliter; CI, confidence interval; ELISA, enzyme-linked immunosorbent assay; SARS-CoV-2, severe acute respiratory syndrome-associated coronavirus 2; S1, spike protein subunit S1

[a]Borderline results were considered positive.
